# Cardiac Mechanics Evaluation in Preschool-Aged Children with Preterm Birth History: A Speckle Tracking and 4D Echocardiography Study

**DOI:** 10.3390/jcm13102762

**Published:** 2024-05-08

**Authors:** Federica Savio, Domenico Sirico, Giada Mazzon, Luca Bonadies, Silvia Guiducci, Daniel Nardo, Sabrina Salvadori, Martina Avesani, Biagio Castaldi, Eugenio Baraldi, Giovanni Di Salvo

**Affiliations:** 1 Neonatal Intensive Care Unit, Department for Women and Children’s Health, University Hospital of Padova, 35128 Padua, Italy; federica.savio.2@gmail.com (F.S.); silviaguiducci24@gmail.com (S.G.); daniel.nardo@aopd.veneto.it (D.N.); sabrinasalvadori27565@gmail.com (S.S.); eugenio.baraldi@unipd.it (E.B.); 2 Pediatric and Congenital Cardiology Unit, Department for Women and Children’s Health, University Hospital of Padova, 35128 Padua, Italy; giada.mazzon@studenti.unipd.it (G.M.); martina.avesani@aopd.veneto.it (M.A.); b.castaldi@yahoo.it (B.C.); giovanni.disalvo@unipd.it (G.D.S.)

**Keywords:** follow-up, preterm, pediatric cardiology

## Abstract

**Background:** The premature-born adult population is set to grow significantly, and prematurity has emerged as an important cardiovascular risk factor. We aimed to comprehensively assess cardiac mechanics and function in a cohort of ex-preterm preschoolers. **Methods:** Ex-preterm children (<30 weeks of gestation), aged 2 to 5 years, underwent transthoracic 2D, speckle-tracking, and 4D echocardiography. The findings were compared with 19 full-term children. **Results:** Our cohort of 38 children with prematurity history showed a normal morpho-functional echocardiographic assessment. However, compared to controls, the indexed 3D end-diastolic volumes of ventricular chambers were reduced (left ventricle 58.7 ± 11.2 vs. 67.2 ± 8.5 mL/m^2^; right ventricle 50.3 ± 10.4 vs. 57.7 ± 11 mL/m^2^; *p* = 0.02). Left ventricle global and longitudinal systolic function were worse in terms of fraction shortening (32.9% ± 6.8 vs. 36.5% ± 5.4; *p* = 0.05), ejection fraction (59.2% ± 4.3 vs. 62.3% ± 3.7; *p* = 0.003), and global longitudinal strain (−23.6% ± 2.4 vs. −25.5% ± 1.7; *p* = 0.003). Finally, we found a reduced left atrial strain (47.4% ± 9.7 vs. 54.9% ± 6.8; *p* = 0.004). **Conclusions:** Preschool-aged ex-preterm children exhibited smaller ventricles and subclinical impairment of left ventricle systolic and diastolic function compared to term children. Long-term follow-up is warranted to track the evolution of these findings.

## 1. Introduction

A newborn is defined as preterm when birth occurs at a gestational age of less than 37 weeks. While the largest share of premature babies are late or moderately preterm, only 5% are extremely preterm, born in less than 28 gestational weeks (GWs), and about 15% are severely preterm (between 28 and 31 GWs) [1].

In Europe and other high-income countries, preterm delivery rates are generally between 5 and 9% [2], while in the United States, the rate varies by state, ranging between 9 and 13% [3]. From a more global perspective, the burden of prematurity weighs on developing countries, with 15 million preterm births estimated per year [4]. On the other hand, survival from prematurity has significantly improved over time; in fact, in high-income countries, approximately 50% of preterm infants born at 24 GWs survive the neonatal period, rising to 90% at 28 GWs and extending to the vast majority of preterm over 32 GWs [4,5].

Prematurity has recently emerged as a significant risk factor for cardiovascular morbidity and mortality [6], associated with an increased incidence of conditions such as hypertension [7,8], heart failure [9], and cerebrovascular and ischemic heart disease [10]. The latest evidence suggests the existence of a peculiar type of cardiac remodeling termed “preterm cardiomyopathy” [11,12], finding its pathophysiological basis in an early shift of the cardiomyocyte’s growth pattern from the fetal hyperplastic pattern to the neonatal hypertrophic response [13], followed by substantial hemodynamic changes and transitioning from a low-resistance placental circulation to a high-resistance systemic circulation. Furthermore, the preterm myocardium may be exposed to an altered hemodynamic load during the neonatal intensive care unit period. In this setting, cardiomyocytes experience accelerated hypertrophy with increased interstitial myocardial collagen deposition, eventually resulting in left ventricle remodeling [14]. According to a recent meta-analysis [12], which synthesized evidence concerning the structural and functional cardiac alterations from infancy to young adulthood in individuals born prematurely, both systolic and diastolic function exhibited deterioration in preterm-born subjects. Although ejection fraction remained stable during childhood, stroke volume decreased in adulthood. Conversely, diastolic dysfunction appeared to worsen over time, accompanied by increased filling pressures since infancy. Right ventricular longitudinal strain was consistently diminished across all preterm groups regardless of age, showing a reduction gradient proportional to prematurity severity. Morphologically, the preterm heart exhibits a persistent decrease in left ventricular chamber volume and tends to experience increased indexed left ventricular mass compared to controls over time. Furthermore, irrespective of prematurity, intrauterine growth restriction (IUGR), leading to fetal flow redistribution, emerges as a potential risk factor for cardiovascular alterations. Indeed, some studies [15,16] indicate that alterations in cardiac geometry persist into preschool and the preadolescent period, resulting in a smaller and more globular heart with compromised longitudinal motility and relaxation.

Several studies have investigated cardiac function in the preterm population in the first months [17,18,19,20], at one year [21,22], at two years [23], and at school age [20,24,25,26]. However, there is limited literature available that compares echocardiographic assessments between preschool-aged children with a history of prematurity and those born at full term. Considering the general increasing trend of preterm birth [27,28], together with an improvement in perinatal care [29], the population of adults born prematurely is expected to expand considerably in the foreseeable future. Consequently, these factors become increasingly pertinent within the realm of public health.

Speckle tracking echocardiography (STE) is an echocardiographic technique whose clinical application has increased exponentially since its first introduction in 2004 [30,31]. This advanced, noninvasive imaging modality allows rapid and accurate assessment of the atrial and ventricular chambers’ global and regional strain, serving as a valuable indicator of diastolic or systolic function.

On the other hand, 3D echocardiography is poised to address an important limitation of 2D echocardiography, which is the exploration of a cardiac chamber through a limited number of tomographic planes, thereby hindering the comprehensive characterization of both morphology and function in a three-dimensional structure. Unlike 2D echocardiography, which relies on geometric assumptions, 3D echocardiography enables direct assessment of right ventricular volumes. These measurements have been validated against MRI, the gold standard, resulting in more precise assessments than those obtained through two-dimensional evaluations [32,33]. With adequate experience in 3D acquisition and post-processing, 3D analysis can easily be incorporated into pediatric standard echocardiographic protocol, clinical assessments, and pediatric patient management [32,33,34]. Furthermore, 3D evaluation could provide a feasible and valid alternative for patients who cannot undergo cardiac magnetic resonance and/or require close cardiological follow-up [32]. Finally, the method currently used should be more appropriately addressed as “4D or real-time 3D echocardiography” since in addition to the three spatial planes (x, y, and z axis), time is considered a “fourth dimension”. In fact, the software does not create a static three-dimensional volume but rather a model that changes over time [35].

## 2. Aim of the Study

Our primary objective was to investigate the presence of significant alterations in mechanics and cardiac function by performing a complete echocardiographic evaluation in a cohort of ex-preterm children around preschool age when compared to controls with similar corrected age and born at term.

The exploratory aim of the study was the analysis of possible risk factors acting in fetal, perinatal, or neonatal cardiovascular development such as IUGR, long exposure to patent ductus arteriosus, inotropic support during the hospital stay, and the diagnosis of BPD.

## 3. Materials and Methods

This is a single-center, prospective observational study. Patients were recruited as part of the outpatients’ follow-up of our neonatal intensive care unit (NICU) from February 2022 to October 2022. The study was conducted according to the guidelines of the Declaration of Helsinki and approved by the Ethics Committee of Padova (protocol code 38n/AO/20, date 20 October 2020), and patients’ enrollment was managed according to the principles of the Helsinki Declaration of 1964 and its subsequent revisions. Neonatological follow-up evaluations, with the aim to monitor growth and neurodevelopment, were scheduled from one month after discharge until the child reached 4–5 years of age. These assessments were planned at intervals of 1, 3, 6, and 12 months, followed by yearly evaluations thereafter. The scheduling took into account presented comorbidities and the corrected age of the child. The medical history data of each patient were obtained through our computerized medical record in use at the Padua University Hospital and by consulting discharge summaries and follow-up reports, thus allowing the selection of patients and the database compilation. Patients’ enrollment was consecutive, applying all the following inclusion criteria to outpatients scheduled for the neonatological follow-up evaluation: age between 2 and 5 years; gestational age at birth ≤30 weeks; previous stay in our NICU; and parental consent. Exclusion criteria were the lack of parental consent; the unavailability of echocardiographic operators; the diagnosis of congenital heart disease or other major malformations or genetic syndromes; and an unsatisfactory quality of imaging due to poor patient compliance. At the same time, a control arm consisting of children with the same corrected age, born at full term, and lacking significant comorbidities was enrolled in a case–control ratio of 2:1, ensuring a balanced male-to-female ratio compared to the cases, following parental consent.

Echocardiographic images were acquired using Philips EPIQ 7C ultrasound machine (Philips, Amsterdam, The Netherlands) and digitally archived in the comPACS software version 10.8 (MediMatic, Genova, Italy). The examination was performed in most cases by a senior pediatric resident (F.S.), under the supervision of an experienced pediatric cardiologist (D.S.) or by the pediatric cardiologist himself.

Conventional echocardiographic evaluation was associated with strain analysis by speckle tracking echocardiography (STE). As regards the conventional echocardiographic evaluation, we performed a complete standardized assessment of the atria and ventricles’ diameters/areas/wall thickness. The ejection fraction (EF) was estimated with Simpson’s single plane method, whereas the LV stroke volume was calculated through LVOT and VTI on the left ventricular outflow. Concerning the evaluation of deformation parameters of the left ventricle, the images were acquired in apical 4-chamber, 3-chamber, and 2-chamber views to evaluate the longitudinal strain (A4C GLS, A3C GLS, A2C GLS, and mean GLS) [36,37]. Furthermore, images were acquired in an apical 4-chamber view focused on the right ventricle and then on the left atrium, respectively, for the evaluation of the right ventricle free wall (RVFWLS) and four chambers (RV4CLS) and the left atrium (LASr-AC) longitudinal strains. Deformation measurements were directly analyzed by the ultrasound machine software, which semi-automatically traces the region of interest (ROI), recognizing the endocardial margin and then allowing the operator to perform adjustments.

The 3D/4D echocardiographic evaluation was performed through the X5-1 probe (Philips), and the acquired images were subsequently subjected to post-processing operations by the echocardiographer on the ultrasound machine. The machine requires the operator to orient a line representing the ventricular longitudinal axis by identifying the apex and atrioventricular valve plane of both ventricles in two orthogonal planes, the corresponding apical 4-chamber and apical 2-chamber views. The system then produces an initial analysis that can be reviewed by the operator, who is invited to better define the margin of the septal wall and the ventricular free wall, evaluated both in long and short axis, throughout the end-diastolic and end-systolic phase. The final processing provides end-diastolic and end-systolic volumes, stroke volume, and right ventricular ejection fraction. Jointly, a dynamic three-dimensional model (therefore 4D) of the right ventricle is produced, which can be explored on the three spatial planes. Moreover, through the same 4D acquisition, the machine software automatically provides further right ventricle parameters, such as ventricular 2D dimensions, TAPSE, septal and free wall strain, and FAC.

Regarding the 4D evaluation of left ventricle volume, probably because of the patients’ small heart size, the vendor software recognizes the left ventricular and atrial structures inaccurately. Therefore, we used “off label” the same process by which right volumes were calculated, “tricking” the software by pointing to the left ventricle instead of the right ventricle and then manually refining the identified margins.

### Statistical Analysis

Statistical data were analyzed using the following programs: MedCalc 19.2.6 (MedCalc Software Ltd., Ostend, Belgium), Excel version 2403 (Microsoft, Redmond, WA, USA), and SPSS 21.0 (IBM, Armonk, NY, USA). The categorical variables were expressed in absolute number and in percentage and then analyzed using Fisher’s exact test, considering a significance level (*p* value) lower than or equal to 0.05. For continuous variables, mean and standard deviation were calculated. In the case of variables with normal distribution, statistical analysis was performed using the Student’s *t*-test for comparison between the values detected in two independent samples. For variables with non-normal distribution, evaluation was performed through non-parametric tests (Mann–Whitney *U*-test). In both cases, a significance level of less than or equal to 5% (*p* value ≤ 0.05) was considered statistically significant.

## 4. Results

### 4.1. Population

**Figure 1:**
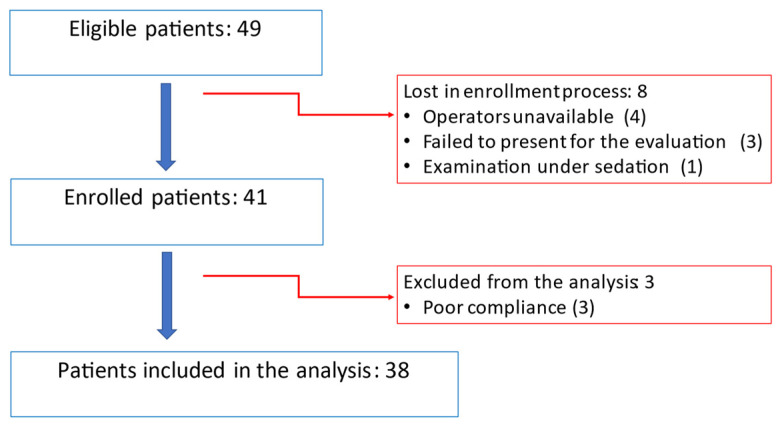
Eligibility and enrollment flow chart.

From 1 February to 25 October 2022, 49 patients met the inclusion criteria, thus being eligible for the study. Unfortunately, we were unable to enroll eight patients due to the following reasons: four patients due to staff unavailability, one patient who already had an echocardiographic evaluation scheduled under sedation, and three patients who did not attend their scheduled evaluations. Additionally, three more cases were excluded from the analysis because of extreme opposition to the examination, leading to 38 patients finally included in the analysis. The eligibility and enrollment process are shown in Figure 1.

Finally, it should be noted that in some cases, it was not possible to collect all the echocardiographic parameters because of suboptimal compliance during the evaluation or inadequate acoustic window for the 4D evaluation. Likewise, blood pressure could not be obtained in all patients.

The demographic and anthropometric characteristics of the 38 cases and of the 19 controls are reported in Table 1.

We found a clear male prevalence; the cases’ average gestational age is less than 27 GW, with a mean birth weight of 815 g. One-third of these patients had a history of intrauterine growth restriction, and 26% were born with a birth weight below the 10th percentile, also known as small for gestational age (SGA). At the time of the evaluation, ex-preterm children had reached a corrected average age of 32 months (2 years and 8 months), comparable to controls. The weight, height, and BSA of premature patients at the time of evaluation tended to be lower than controls, but only weight difference was statistically significant. As regards the vital parameters, heart rate and blood pressure did not differ between the two groups.

Regarding respiratory support parameters, reported in Table 2, all patients underwent non-invasive ventilation, and nearly 75% of them required invasive ventilation during their NICU stay. Not surprisingly, 61% of these patients were diagnosed with BPD at 36 GW according to the Jensen criteria [38]. The great majority underwent surfactant administration. At follow-up after discharge, half of these children presented episodes of bronchial hyperreactivity, and one-third required hospitalization for respiratory disease.

With respect to cardiovascular history, as shown in Table 2, only 5% exhibited documented persistent pulmonary hypertension (PPHN) at the echocardiographic evaluation. A hemodynamically significant ductus arteriosus, defined on the echocardiographic assessment by a diameter > 1.5 mm or 1.4 mm/kg and unrestrictive doppler pattern, was found in two-thirds of the cases and treated with paracetamol or ibuprofen. Only in three cases (8%) did medical therapy fail, and therefore, surgical ligation was performed. At discharge, no one had systemic or pulmonary arterial hypertension; no cardiological therapy was administered. Additional comorbidities are also highlighted in Table 2.

### 4.2. Echocardiographic Assesment

**Figure 2:**
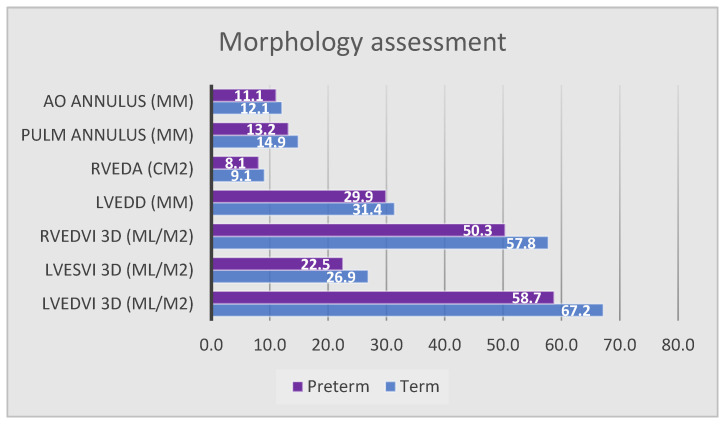
Echocardiographic morphologic parameters, significant results. Data reported as mean. LVEDD, left ventricle end-diastolic diameter; LVEDVi, indexed left ventricle end-diastolic volume; LVESDi, indexed left ventricle end-systolic diameter; LVESVi, indexed left ventricle end-systolic volume; RVEDA, right ventricle end-diastolic area; RVEDVi, indexed right ventricle end-diastolic volume.

**Figure 3:**
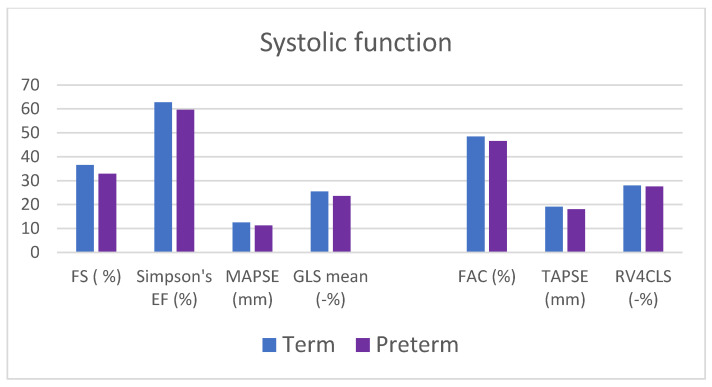
Main echocardiographic functional parameters. Data reported as mean. EF, ejection fraction; FAC: fractional area change (auto when calculated through 3D software QLAB 10 integrated in the ultrasound machine); FS, fractional shortening; GLS, global longitudinal strain; MAPSE, mitral annular plane systolic excursion; RV4CLS, right ventricle 4-chamber longitudinal strain; TAPSE, tricuspid annular plane systolic excursion.

Our cohort of patients with prematurity history showed regular morphological and functional echocardiographic assessment. No one presented pulmonary hypertension. However, when compared to full-term born controls, some statistically significant differences emerged (Figure 2 and Figure 3; Table 3 and Table 4).

#### 4.2.1. Morphologic Assessment

Regarding morphologic assessment, as demonstrate in Table 3 and Figure 2, left ventricle posterior wall thickness in systole (LVPWs, 6.7 mm ± 1 vs. 8.2 mm ± 1.2; *p* < 0.001), left ventricle end-diastolic diameter (LVEDD 29.9 mm ± 2.2 vs. 31.4 mm 2.6; *p* = 0.024), and aortic (11 mm ± 1 vs. 12.1 mm ± 0.9; *p* < 0.001) and pulmonary valve annulus (13.2 mm 1.7 vs. 14.9 mm ± 1.7; *p* < 0.001) appeared significantly smaller in preterm subjects.

Similarly, 4D analysis showed a reduced right ventricular end-diastolic area (RVEDA, 8.1 cm^2^ ± 1.5 vs. 9.1 cm^2^ ± 1.1; *p* = 0.007) and indexed right ventricular end-diastolic volume (RVEDVi, 50.3 mL/m^2^ ± 10.4 vs. 57.7 mL/m^2^ ± 11; *p* = 0.022). Significantly lower 4D end-systolic and end-diastolic volumes were also obtained for the left ventricle (LVEDVi 58.7 mL/m^2^ ± 11.2 vs. 67.2 mL/m^2^ ± 8.5 *p* = 0.021; LVESVi 22.5 mL/m^2^ ± 7.2 vs. 26.9 mL/m^2^ ± 4.8 *p* = 0.049), while no significant difference was found in 2D volume measurement through Simpson’s method. Coherently with the minor LVEDD, the diameter of the mid-cavity appeared shorter (29.7 mm ± 2.9 vs. 31.4 mm ± 2.2; *p* = 0.025), without differences regarding the sphericity index. Finally, the stroke volume, calculated through LVOT and VTI on the left ventricular outflow, was significantly reduced (17.2 ± 3.8 vs. 20.1 mm ± 4; *p* = 0015). In contrast with the other investigated cardiac chambers, the left atrial area and volume did not prove to be reduced compared to controls.

#### 4.2.2. Functional Assessment

In terms of left ventricular systolic function, fraction shortening (32.9% ± 6.8 vs. 36.5% ± 5.4; *p* = 0.05) and ejection fraction calculated with Simpson’s method (59.2% ± 4.3 vs. 62.3% ± 3.7; *p* = 0.003), although within normal range, were significantly lower in children with a history of prematurity (Figure 3 and Table 4). Left ventricular longitudinal function calculated by MAPSE (11.3 mm ± 2.1 vs. 12.6 mm ± 1.4; *p* = 0.021) and global longitudinal strain (mean GLS −23.6% ± 2.4 vs. −25.5% ± 1.7; *p* = 0.003) were significantly reduced in the ex-preterm group.

Regarding diastolic function, the study of the mitral flow pattern (E/A ratio) together with tissue Doppler (lateral and septal E/e’ ratio) did not show any differences between the two groups. Conversely, the analysis of the left atrial strain revealed a decreased reservoir value in former preterm subjects (47.4% ± 9.7 vs. 54.9% ± 6.8; *p* = 0.004).

The right ventricular systolic function was normal in the two groups without any significant difference found using traditional 2D parameters (FAC and TAPSE), speckle tracking analysis, or at the 4D evaluation (EF, FAC, TAPSE, longitudinal strain free wall, and septum calculated automatically). Similarly, no pulmonary hypertension parameter was highlighted; there were no differences in terms of esteemed right ventricle pressure through tricuspid regurgitation and interventricular septal morphology evaluation or in pulmonary resistance appraised by pulmonary artery acceleration time (PAAT) and its ratio with ejection time (PAAT/ET).

### 4.3. IUGR and SGA

In our cohort, 11 patients were SGA (29%), and 12 infants were IUGR (32%). IUGR patients demonstrated a persistently lower weight compared to non-IUGR preterms (11.2 kg ± 1.2 vs. 12.8 kg ± 2.4; *p* = 004). Regarding echocardiographic parameters, IUGR premature subjects, with respect to non-IUGR ones, showed an increased indexed volume of the left atrium (19.3 mL/m^2^ ± 4.2 vs. 14.4 mL/m^2^ ± 5.1; *p* = 0.01) and a shorter, more globular left ventricle morphology with a sphericity index closer to 1 (1.5 ± 0.2 vs. 1.7 ± 0.2; *p* = 0.01), which was confirmed at multivariate analysis. On the other hand, no differences were found in terms of ventricular function parameters and signs of pulmonary hypertension.

### 4.4. Previous NICU Stay

Patients who received inotrope therapy during their NICU stay had an average lower gestational age (25.6 GW ± 1.5 vs. 27.4 GW ± 1.8; *p* = 0.003) and presented, overall, a more complex hospitalization, prolonged invasive ventilation and a higher rate of BPD diagnosis, increased incidence of IVH, hemodynamically significant ductus arteriosus (DA), and DA surgical ligation. Comparing the group of patients who required inotropes to those who did not, we could not identify any statistical difference among morphologic and functional parameters at standard and advanced echocardiographic evaluation.

Similarly, as regards the persistence of hemodynamically significant DA, defined as the need for at least three pharmacological cycles or surgical ligation to achieve closure, no statistically significant difference was found among the studied echocardiographic parameters.

### 4.5. BPD

Comparing the data of BPD patients with that of those without a diagnosis of BPD, we did not find any statistically significant difference regarding the morphological assessment, although there is a tendency of reduced biventricular volumes at 4D evaluation.

Conversely, from a functional point of view, in patients with BPD, TAPSE appears to be significantly reduced (17.2 mm ± 2.4 vs. 18 mm ± 5.4; *p* = 0.02), and so does the TAPSE/PASP ratio (0.67 mm/mmHg ± 0.14 vs. 0.78 mm/mmHg ± 0.3; *p* = 0.01).

## 5. Discussion

The present study investigated cardiac morphologic and functional status in a group of preschool age children with a history of prematurity and subsequently compared the results with full-term children of the same corrected age. Our findings are the result of standard 2D echocardiography and more advanced echocardiographic techniques, such as STE and 4D evaluation, which have been scarcely reported in this population previously, making it difficult to compare our results with previous works (Table 5).

Regarding cardiac geometry, a reduction in LA, aortic valve root, and both ventricular chambers’ size parameters is a common finding in other studies performed at school age [25,26], while a smaller pulmonary valve annulus was not highlighted previously. Conversely, Erickson’s group [21] showed an increase upon 2D evaluation in right ventricular dimensions at 1 year of life.

Four-dimensional assessment of ventricular size and shape has never been investigated previously in this specific population. Regarding this novel evaluation technique, our data confirm the tendency towards a reduced growth of the ventricular chambers in the preterm group.

With respect to right ventricular function, our study did not bring to light any significant differences between preterm and control groups in terms of longitudinal (TAPSE and RV longitudinal strain) and global systolic function (FAC and 3D EF). Two studies showed a reduction in the longitudinal strain of the right ventricle at 1 and 2 years of life [21,23], but only one [21] found a reduction in FAC and TAPSE. Possibly, the absence of cases of pulmonary hypertension together with the longer temporal distance from the neonatal period in our cohort may have influenced this variable. In fact, in both the aforementioned studies, PAAT and PAAT/ET were significantly reduced, thus suggesting a study population with worse pulmonary vasculature disease. In accordance with our experience, another study [20] did not find differences in PVR at 7 years of age.

Left ventricular function has been investigated in school-aged ex-preterm children. In agreement with our findings, Mohlkert et al. [25] demonstrated worse systolic function, expressed as a reduction in fraction shortening and in left ventricle longitudinal function (MAPSE and lateral s’ velocity), however, with preserved global longitudinal strain. Regarding diastolic function, an increase in E/lateral e’ ratio was reported in the same study, suggesting worse diastolic performance. Conversely, diastole did not appear altered according to the 81 patients’ data in the study by Kwinta’s group [26]. Standard 2D assessment of diastolic function did not show any alterations in our population. However, left atrial strain assessment has recently emerged as a fine tool for the assessment of LV diastolic function in adult and pediatric populations [39,40], and it represents a novel and previously uninvestigated parameter in cardiologic assessment of ex-preterm patients. Interestingly, our analysis demonstrated a reduced LA strain in ex-preterm children compared to controls.

The analysis of our ex-preterm population based on the history of IUGR displayed that these patients present a more spherical geometry of the left ventricle. The same finding is confirmed by Crispi et al. [16], together with diastole impairment, which was not fully supported in our patients (LV E/e’ tended to be higher in children with IUGR without reaching statistical significance). This impaired function could be secondary to an altered heart development, where cardiomyocytes appear thinner and equipped with shorter sarcomeres [41]. Moreover, the increased afterload, initially because of an elevated placental resistance and, subsequently, due to the fetal peripheral vasoconstriction, can lead to an hypertrophic response in the most severe forms that characterize early IUGRs [42].

Hemodynamically significant DA in preterm patients is thought to be a negative prognostic factor in terms of long-term complications [43,44,45], and many advocate the early closure of DA, either pharmacological or interventional [46]. However, when analyzing our population based on long-term exposure to DA patency, we could not find any significant differences in the echocardiographic assessment between the two groups. This could be attributed to several factors, such as the temporal distance of the event and thus normalization over time, the relatively short temporal exposure to this altered hemodynamic condition, and the paucity of subjects who suffer this condition.

Furthermore, we analyzed ex-preterm children based on the previous diagnosis of BPD. The comparison of echocardiographic assessment in premature infants diagnosed with BPD versus those without BPD has been examined in other studies, and the comparative results are available in Table 6.

Regarding evaluation of the left ventricle, Korhonen’s study [20] is the only one to have demonstrated a morphological change with reduction in IVSd and LVESD. Left ventricle systolic function, on the other hand, was similar at 1 year of life [22] and at school age [20].

Concerning right-sided chambers’ analysis, there is general agreement on worse right ventricular function, whether it is evaluated through TAPSE, FAC, or longitudinal strain [21,22,23]; in our cohort, we observed a significant TAPSE reduction. Ventricular areas and diameters seem to be increased at the assessment of the American series at 1 year of life [21]. Regarding pulmonary resistances, there is conflicting evidence; indeed, some [21,47] found a reduction in the PAAT and PAAT/ET ratio, while other studies [20,24], including ours, did not demonstrate any difference. We evaluated the TAPSE/PASP ratio as a new parameter, recently identified as a prognostic indicator in the field of pulmonary hypertension [48]. Interestingly, in our cohort, children with BPD had a significantly reduced, although within the normal range, TAPSE/PASP ratio compared to non-BPD preterms.

The strengths of our study are undoubtedly the evaluation of a patient subgroup that has been poorly studied in the literature, for which there is still no well-defined evidence; moreover, the matching with controls was very accurate. Our patients underwent an extensive and advanced, comprehensive echocardiographic evaluation of new and promising parameters still scarcely considered in this research area, such as right and left ventricle 4D evaluation, atrial strain, and TAPSE/PASP ratio.

However, regarding limitations, the sample size is relatively small; therefore, the series deserves to be expanded in the upcoming years. Additionally, we acknowledge intrinsic limitations of 2D and especially 3D/4D and the speckle tracking technique depending on good image quality and the need for high frame rates, operator experience, loading conditions, and inter-vendor variability [49,50,51]. With the data available, further post-processing studies such as radial and circumferential strain and myocardial work might be evaluated to further enrich our work.

## 6. Conclusions

In conclusion, our study revealed morphologic peculiarities and subclinical impairments of systolic and diastolic function at cardiologic follow-up in preschool children with prematurity history when compared with a control group of children born at term.

The hearts of these children appeared to be characterized by reduced biventricular volumetry. Systolic and diastolic function of the left ventricle, although within the normal range, showed an overall worse performance compared to controls. Long-term follow-up is warranted to assess the evolution of these findings.

## Figures and Tables

**Table 1:** General, demographic data, and vital and anthropometric parameters.

	Preterms	Controls	*p* Value
Number of patients	38	19	
Female	12 (32%)	6 (32%)	1
Gestational age at birth (GW)	26.7 + 1.87	39.9 + 1.3	
Birth weight (g)	815 + 232	3359 + 413	
SGA	11 (29%)	3 (18%)	0.28
IUGR	12 (32%)	1 (6%)	0.026 *
Chronological age (months)	34.9 + 9.4	32.2 + 7.1	0.26
Corrected age (months)	31.9 + 9.1	32.2₊7.1	0.9
Weight (kg)	12.2 + 2.2	13.6 + 1.8	0.029 *
Height (cm)	90.1 + 6.5	93.6+ 6.5	0.074
BSA (cm^2^)	0.56 + 0.1	0.59 + 0.1	0.085
Heart rate (bpm)	107 + 20	107 + 11	0.96
SBP (mmHg)	103 + 11	99 5	0.24
DBP (mmHg)	65 + 10	66 + 9	0.71

Data reported as frequency (%), median +/− IQR and mean +/− SD. * *p* < 0.05. BSA, body surface area; DBP diastolic blood pressure; GW, gestational weeks; IUGR, intrauterine growth restriction; SBP, systolic blood pressure; SGA, small for gestational age.

**Table 2:** Medical history and main comorbidities of the preterm cohort.

Categorical Variables		n°	%
Respiratory	Need for mechanical ventilation	38	100
	Invasive mechanical ventilation	28	74
	Need for surfactant administration	32	84
	Technique: LISA	9	28
	InSurE	1	3
	Infant intubated	21	66
	Mixed	1	3
	BPD at 36 weeks (PMA)	23	61
	Discharged with O_2_ supplementation	5	13
	Diuretic therapy at discharge	10	26
	Broncho-reactivity	19	50
	Admissions for respiratory disease	13	33
Cardiovascular	Persistent pulmonary hypertension in newborn	2	5
	Inotropic support	15	39
	Hemodynamically significant DA Surgical legation	25 3	66 8
Other comorbidities	IVH > I grade	7	18
	Chronic renal failure	2	5
	NEC Surgery needed	9 5	24 13
Continuous variables		mean ± SD	
Respiratory	Invasive mechanical ventilation (days)	14.4 ± 6	
	Non-invasive mechanical ventilation (days)	51.3 ± 19.8	
	Number of surfactant administrations (n°)	1.3 ± 0.9	
Cardiovascular	Inotropic support (days)	2.1 ± 3.5	
	Medical cycles to treat ductus arteriosus (n°)	1.7 ± 1.4	

Data reported as absolute number, frequency (%) and mean +/− SD. BPD, bronchopulmonary dysplasia; DA, ductus arteriosus; InSurE, intubation, surfactant, and extubation; IVH, intraventricular hemorrhage; LISA, less invasive surfactant administration; NEC, necrotizing enterocolitis; PMA, post-menstrual age.

**Table 3:** Echocardiographic morphologic parameters.

Echocardiography Parameters	Cases Mean ± SD	Controls Mean ± SD	*p*-Value
Left-sided heart:			
IVSd (mm)	4.0 ± 0.4	3.9 ± 0.4	0.56
LVEDD (mm)	29.9 ± 2.2	31.4 ± 2.6	0.024 *
PWd (mm)	5.1 ± 0.8	5.0 ± 0.8	0.63
IVSs (mm)	5.7 ± 1.1	5.6 ± 0.7	0.95
LVESD	20.0 ± 2.5	19.9 ± 1.4	0.75
PWs (mm)	6.7 ± 1	8.2 ± 1.2	<0.001 *
Mid-cavity diameter (mm)	29.7 ± 2.9	31.4 ± 2.2	0.025 *
Sphericity index	1.64 ± 0.24	1.58 ± 0.1	0.26
LVEDV 3D (mL)	33.5 ± 7.8	39.8 ± 5.2	0.011 *
LVEDVi 3D (mL/m^2^)	58.7 ± 11.2	67.2 ± 8.5	0.021 *
LESV 3D (mL)	12.9 ± 4.3	15.9 ± 3.1	0.024 *
LVESVi 3D (mL/m^2^)	22.5 ± 7.2	26.9 ± 4.8	0.049 *
Aortic annulus diameter (mm)	11 ± 1	12.1 ± 0.9	<0.001 *
Right-sided heart:			
RVEDA (cm^2^)	8.1 ± 1.5	9.1 ± 1.1	0.007 *
RVESA (cm^2^)	27.5 ± 3.6	28 ± 2.4	0.59
RVEDV 3D (mL)	28.1 ± 8	34.3 ± 7.9	0.011 *
RVEDVi 3D (mL/m^2^)	50.3 ± 10.4	57.7 ± 11	0.022 *
RVESV 3D (mL)	11.8 ± 4.6	13.0 ± 3.0	0.35
RVESVi 3D (mL/m^2^)	21.1 ± 6.7	21.8 ± 4.3	0.68
Pulmonary annulus diameter (mm)	13.2 ± 1.7	14.9 ± 1.7	<0.001 *

Data reported as mean +/− SD. * *p* < 0.05. IVSd-s, interventricular septum in diastole-systole; LVEDD, left ventricle end-diastolic diameter; LVEDV, left ventricle end-diastolic volume (-i if indexed); LVESD, left ventricle end-systolic diameter; LVESV, left ventricle end-systolic volume (-i if indexed); PWd-s, posterior wall in diastole-systole; RVEDA, right ventricle end-diastolic area; RVESA, right ventricle end-systolic area; RVEDVright ventricle end-diastolic volume (-i if indexed); RVESV, right ventricle end-systolic volume (-i if indexed).

**Table 4:** Echocardiographic functional parameters.

Echocardiography Parameters	Cases Mean ± SD	Controls Mean ± SD	*p*-Value
Left ventricle systolic function:			
Fraction shortening (%)	32.9 ± 6.8	36.5 ± 5.4	0.049 *
Ejection fraction (Simpson single plane) (%)	59.2 ± 4.3	62.3 ± 3.7	0.003 *
Stroke volume (mL)	17.2 ± 3.8	20.1 ± 4	0.015 *
MAPSE (mm)	11.3 ± 2.1	12.6 ± 1.4	0.021 *
4-chamber GLS (%)	−23.7 ± 2.8	−26 ± 2.5	0.004 *
3-chamber GLS (%)	−23.3 ± 3.3	−25.4 ± 3.1	0.027 *
2-chamber GLS (%)	−23.8 ± 3.3	−25.0 ± 2.6	0.16
GLS mean (%)	−23.6 ± 2.4	−25.5 ± 1.7	0.003 *
Left ventricle diastolic function:			
Atrial Strain (%)	47.4 ± 9.7	54.9 ± 6.8	0.004 *
E/A	1.7 ± 0.4	1.9 ± 0.5	0.25
E/e’ lateral	6.6 ± 1.3	6.3 ± 1.4	0.38
E/e’ septal	8.4 ± 1.7	8.4 ± 1.2	0.99
RV Systolic function:			
TAPSE (%)	18.0 ± 2.6	19.1 ± 2.4	0.13
TAPSE auto 3D (%)	15.8 ± 3	16.9 ± 1.8	0.17
FAC (%)	46.6 ± 7.4	48.4 ± 6.1	0.36
FAC auto 3D (%)	49.5 ± 8.6	50.9 ± 5.1	0.55
RVFWLS (%)	−32.8 ± 5	−33.5 ± 3.4	0.61
RV4CLS (%)	−27.5 ± 3.6	28.0 ± 2.4	0.59
Auto septal RVLS (%)	−21.9 ± 5.8	−22.8 ± 4.3	0.53
Auto free wall RVLS (%)	−29.4 ± 6	−32.3 ± 3.6	0.08

Data reported as mean +/− SD. * *p* < 0.05. FAC, fractional area change (auto when calculated through 3D software); GLS, global longitudinal strain; MAPSE, mitral annular plane systolic excursion; RV4CLS, right ventricle 4-chamber longitudinal strain; RVFWLS, right ventricle free wall longitudinal strain; RVLS, right ventricle longitudinal strain. TAPSE, tricuspid annular plane systolic excursion (auto when calculated through 3D software).

**Table 5:** Comparison of echocardiographic parameters of patients with a history of prematurity compared with infants born at term. Sample size, age of patients, and inclusion criterion of prematurity/birth weight are reported.

Preterm vs. Term Children	Erickson 80 pt 1 y < 29 GWs		Kang 24 pt 2 y < 33 GWs	Mohlkert 176 pt 6 y < 26 GWs		Korhonen 34 pt 7.5 y < 1500 g		Kwinta 81 pt 7 y ELBW		Savio 38 pt 2–5 y < 30 GWs	
Right sections	Areas and diameters ↑							RVEDD ↓		RVEDA RVEDV 3D Pulmonary annulus	↓
MMODE											
Areas											
Volume 3D											
RV function	TAPSE, FAC, FWLS ↓		FAC = strain ↓							TAPSE, FAC, strains, EF 3D	=
TAPSE, FAC											
strain											
EF 3D											
RV afterload	PAAT; PAAT/ET	↓	RVSP ↑ PAAT, mPAP =			PAAT; PAAT/ET	=			PAAT; PAAT/ET RVSP	=
PAAT, PAAT/ET											
RVSP											
Left sections				LV lenght, mid cavity, Ao Annuls, mass Stroke volume	↓			PWs, LVEDD Annulus Ao Simpson volumes Stroke volume	↓	PWs, LVEDD, mid cavity, Ao annulus, 3D volumes stroke volume	↓
MMODE											
Simpson											
3D											
LV function				MAPSE, FS, s’ ↓ GLS = E/e’ lat ↑				Diastolic function =		GLS, EF, FS Left atrial strain	↓
GLS, FS, EF											
MAPSE, s’											
diastolic											
Left atrium				Dimensions =				Diameter ↓		Dimensions =	

EF, ejection fraction; ET, ejection time; FAC, fractional area change (auto when calculated through 3D software); FS, fractional shortening; FWLS, free wall longitudinal strain. GLS, global longitudinal strain; LV, left ventricle; MAPSE, mitral annular plane systolic excursion; mPAP, mean pulmonary artery pressure; PAAT, pulmonary artery acceleration time; PWs, posterior wall in systole; RV, right ventricle; RVEDA, right ventricle end-diastolic area; RVEDV, right ventricle end-diastolic volume; RVSP, right ventricle systolic pressure; TAPSE, tricuspid annular plane systolic excursion. Up arrow (↑) for increased values, down arrow (↓) for decreased values, equal symbol (=) for no difference.

**Table 6:** Comparison of echocardiographic parameters of premature patients diagnosed with BPD versus premature patients without BPD. Sample size, age of patients, and inclusion criterion of prematurity/birth weight are reported.

Preterm with BPD vs. Preterm without BPD	Erickson 80 pt 1 y < 29 GWs		Levy 239 pt 1 y < 29 GWs (BPD e/o PH)	Kang 24 pt 2 y < 33 GW	Koroglu 41 pt 2–4 y		Joshi 60 pt 8–12 y < 32 GWs		Korhonen 34 pt 7–8 y < 1500 g		Savio 38 pt 2–45 y < 30 GWs	
LV function			GLS =						FS =		=	
GLS												
EF; FS												
RV function	TAPSE, FAC, FWLS	↓	Strain ↓	Strain ↓							TAPSE ↓	
TAPSE												
FAC												
strain												
RV afterload	PAAT; PAAT/ET	↓			PAAT; PAAT/ET	↓	PAAT; PAAT/ET	=	PAAT; PAAT/ET	=	PAAT;	=
PAAT											PAAT/ET	
PAAT/ET											TAPSE/PASP	↓
RV dimension	Areas Diameters	↑									=	
Areas												
3D volumes												
LV dimension									IVSd LVESD	↓	=	
Volumes												
MMODE												

EF, ejection fraction; ET, ejection time; FAC, fractional area change (auto when calculated through 3D software); FS, fractional shortening; FWLS, free wall longitudinal strain; GLS, global longitudinal strain; IVSd, interventricular septum in diastole; LV, left ventricle; LVESD, left ventricle end-systolic diameter; PAAT, pulmonary artery acceleration time; PASP, pulmonary artery systolic pressure; RV, right ventricle; TAPSE, tricuspid annular plane systolic excursion. Up arrow (↑) for increased values, down arrow (↓) for decreased values, equal symbol (=) for no difference.

## Data Availability

The original contributions presented in the study are included in the article, further inquiries can be directed to the corresponding author.
